# The Influence of Organofunctional Substituents of Spherosilicates on the Functional Properties of PLA/TiO_2_ Composites Used in 3D Printing (FDM/FFF)

**DOI:** 10.3390/polym14245493

**Published:** 2022-12-15

**Authors:** Bogna Sztorch, Daria Pakuła, Magdalena Kustosz, Eliza Romanczuk-Ruszuk, Ewa Gabriel, Robert E. Przekop

**Affiliations:** 1 Centre for Advanced Technologies, Adam Mickiewicz University in Poznań, 10 Uniwersytetu Poznańskiego, 61−614 Poznań, Poland; 2 Faculty of Chemistry, Adam Mickiewicz University in Poznań, 8 Uniwersytetu Poznańskiego, 61−614 Poznań, Poland; 3 Institute of Biomedical Engineering, Faculty of Mechanical Engineering, Bialystok University of Technology, Wiejska 45C Street, 15−351 Bialystok, Poland

**Keywords:** PLA, POSS, polylactide, silsesquioxane, 3D printing, additive manufacturing, titanium dioxide

## Abstract

In this study, the influence of TiO_2_ pigment filler modified with spherosilicate derivatives on the processes and thermomechanical properties of composites based on PLA was investigated. Rheological tests (MFR) were carried out, on the basis of which it was found that the addition of organosilicon compounds has a plasticizing effect on the polymer-filler systems. TGA and DSC analysis were performed. The analysis of the contact angle showed that 1.5% of the additives had an influence on the superhydrophobic properties of TiO_2_ (above 135°), and a slight improvement of this parameter was also observed for composites containing the modified pigment. Microscopic analysis and mechanical tests (tensile strength, impact strength and flexural strength tests) were carried out as well. It has been observed that the addition of certain derivatives adversely affects the dispersion of the filler, thus a slight improvement in mechanical properties is observed. For modifiers that do not affect filler agglomeration, a plasticizing effect on the composite is observed.

## 1. Introduction

In recent years, there has been a noticeable interest in additive techniques, especially in 3D printing. This technology is based on dividing the model into layers, which are then printed layer by layer. The advantage of 3D printing is the ability to produce complex parts, the lower cost and reduced production time [1,2,3]. In the fused deposition modeling (FDM) method, layers of material are created by heating a thermoplastic fiber, which is extruded from a die and deposited on a platform. The properties of the elements produced by the FDM method depend on the process parameters, such as: layer height, nozzle diameter, filling gap, structure direction, filling angle, number of contours, printing speed and extruder temperature [1,4].

Polylactic acid (PLA) is one of the most popular biodegradable polymers based on natural resources [5,6]. As mentioned, PLA is a biodegradable material and it degrades into lactide. It is characterized by relatively good mechanical properties, durability, being environmentally friendly and ease of processing, therefore it is currently a popular material in many scientific studies [7,8,9]. In addition, PLA is characterized by high transparency, comparable to PS and PET, and low toxicity, which is why it is used in many fields, e.g., as a material for biomedical applications and food packaging [10,11,12]. In specialized applications, the properties of PLA are unsatisfactory, especially its low impact strength or adjusted thermal conductivity [5,13]. The poor properties of PLA limit the use of this material in many industries. Therefore, a lot of scientific research focuses on improving them. The properties of PLA can be upgraded by adding suitable modifiers such as plasticizers or fillers, such as particles and fibers (e.g., glass fiber or carbon fiber) [14,15].

There are many studies on the features of PLA-based composites. To increase the thermomechanical stability or to introduce the electrical conductivity of PLA graphene-based materials are added [16,17]. Another example is to blend other polymers with PLA to produce a mechanically more stable material [16,18,19]. To use PLA in certain industries, such as biomedical engineering or agricultural transport, it must have shown antimicrobial properties. For this purpose, TiO_2_, ZnO or other ceramic materials are added to the PLA as is shown in other publications [20,21,22]. Titanium dioxide especially is used as a filler for PLA-based composites for industrial applications. It is a biocompatible material with high thermal stability, and is also used as a pigment additive in the whitening of plastics. This oxide is characterized by a high refractive index; therefore, it can be used as anti-reflective coatings in optics [23].

In recent years, there has been a noticeable interest in the use of spherosilicates and silsesquioxanes and their derivatives as modifiers in composite materials [24]. A great influence of derivatives of these compounds, used as modifiers, on the properties of composite materials was observed. This may be related to the fact that after introducing the modifier into the polymer matrix, the inorganic core of the spherosilicate/silsesquioxane cage provides molecular enhancement, and the organic groups increase the compatibility with the polymer matrices [25]. Zhang F. et al. [26] examined how the material coated with oligomeric silsesquioxane was characterized by high corrosion resistance. Many studies have noted an improvement in mechanical properties after adding spherosilicates [27,28,29]. It has been observed that the addition of silsesquioxanes in the range of 1–3 wt.% can increase the crystallinity degree and the mechanical properties of the composites [25,30]. Furthermore, these modifiers have a positive effect on the rheological properties and thermal stability of these materials [31,32,33].

The aim of the work was to investigate the influence of organofunctional spherosilicate substituents on the processing and properties of PLA/TiO_2_ composites dedicated for use in 3D printing, FDM/FFF technique. Three-dimensional (3D) printing, which is a processing technique classified as micro-extrusion, poses different process requirements for materials compared to classic extrusion. The scale of the tool is 10 to 30 times smaller than in industrial extruders. For this reason, the MFR parameter is one of the most important for the correctness of the printout. Titanium dioxide is the basic pigment and color carrier for thermoplastic systems. However, due to its hydrophilic nature, surface modifications are necessary to increase compatibility with the polymer matrix.

## 2. Materials and Methods

### 2.1. Materials

Polylactide (PLA) Ingeo 2003D type was purchased from NatureWorks (Minnetonka, MN, USA). TiO_2_ was obtained from Grupa Azoty Z.Ch. “Police” S.A. (Police, Poland), as a 50% *w*/*w* water slurry with no additives. The chemicals were purchased from the following sources: tetraethoxysilane (TEOS), vinyltrimethoxysilane (VTMOS) from Unisil (Tarnów, Poland), chlorodimethylsilane, tetramethylammonium hydroxide (TMAH) 25% methanol solution from ABCR, *(R)-(+)*-limonene, 1-octadecene, toluene, *D*-chloroform and Karstedt’s catalyst xylene solution from Merck Life Science Sp.z.o.o (Darmstadt, Germany), P_2_O_5_ from Avantor Performance Materials Poland S.A. (Gliwice, Poland) Toluene was degassed and dried by distilling it from P_2_O_5_ under an argon atmosphere.

### 2.2. Analyses

^1^H and ^29^Si Nuclear Magnetic Resonance (NMR) spectra were recorded at 25 °C on a Bruker Ascend 400 and Ultra Shield 300 spectrometers using CDCl_3_ as a solvent. Chemical shifts are reported in ppm with reference to the residual solvent (CHCl_3_) peaks for ^1^H.

Water Contact Angle (WCA) analyses were performed by the sessile drop technique at room temperature and atmospheric pressure with a Krüss DSA100 goniometer. Three independent measurements were taken for each sample, each with a 5 µL water drop, and the obtained results were averaged to reduce the impact of surface nonuniformity.

Thermogravimetry (TGA) was performed using a NETZSCH 209 F1 Libra gravimetric analyzer (Selb, Germany). Samples of 5 ± 0.2 mg were cut from each granulate and placed in Al_2_O_3_ crucibles. Measurements were conducted under nitrogen (flow of 20 mL/min) in the range of 30–800 °C and a 10 °C/min heating rate.

Differential scanning calorimetry (DSC) was performed using a NETZSCH 204 F1 Phoenix calorimeter. Samples of 6 ± 0.2 mg were cut from each granulate and placed in an aluminum crucible with a punctured lid. The measurements were performed under nitrogen in the temperature range of 20 ÷ 220 °C and at a 10 °C/min heating rate.

The effect of the modifier addition on the Mass Flow Rate (MFR) was also determined. The measurements were made using an Instron Ceast MF20 plastometer in accordance to the ISO 1133 standard. The measurement temperature was 210 ± 0.5 °C, while the piston loading was 2.16 kg.

Tensile and Flexural Strength Tests were performed with the Universal testing machine Instron 5969, in accordance to the norm EN ISO 527−2:1996 and ISO 178:2006. The speed of traverse was set to 2 mm/min for both tensile strength and flexural strength tests. Tests on the obtained specimens were performed using a universal testing machine Instron 5969 with a 50 kN maximum load measuring capability.

Charpy impact test was performed on a Instron Ceast 9050 impact machine according to ISO 179−1. For all the series, 7 measurements were performed using standard unnotched samples.

Images of the surface and fractures of the composites were taken using a Keyence VHX-7000 digital microscope (Keyence International, Belgium, NV/SA) with a VH-Z100R wide-angle zoom lens at 1000× magnification and laser confocal scanning microscopy Olympus LEXT OLS 4000 (Tokio, Japan).

The measurements of hiding power were performed by placing the samples of printed parts in the optical path between the LED light source and a UV-NIR spectrophotometer, AvaSpec-Mini2048CL (Avantes, Louisville, CO, USA). The amount of light transmitted through the sample was measured, and on this basis, the relative hiding power was determined, with a 0.5% TiO_2_ PLA sample being used as a reference.

### 2.3. The Procedure for Synthesis of Octaspherosilicate Derivatives

Octahydrospherosilicate (SS) was prepared according to a literature procedure [34]. The procedure of octadecene (8OD) as well as octadecene and vinyltrimethoxysilane (5OD:3VT) derivatives synthesis was descibed in our earlier paper [35].

The procedure of limonene/methoxysilyl derivatives synthesis: In a typical procedure, in a 500 mL three-neck, round-bottom flask 30 g of octahydrospherosilicate, 300 mL of dry toluene and mixture of olefins limonene and vinyltrimethoxysilane in molar ratio 6:2 (24.11 g and 8.75 g) were added (6Limo:2VT).

A magnetic stirring bar was added and a thermometer and condenser equipped with an argon inlet and oil bubbler were attached, the flask placed in a heating mantle and the system was purged with argon. The reaction mixture was set to 110 °C and before reaching boiling point, Karstedt’s catalyst solution (10^−5^ eq. Pt/mol Si-H) was added. The reaction mixture was kept under reflux for 24 h and samples were taken for FT-IR (the progress of the reaction was monitored until full conversion of the Si-H group was observed). Then, the solvent was evaporated under vacuum to dryness to obtain an analytically pure sample.

### 2.4. The Procedure of Mixing TiO_2_ with the Modifier

The procedure of mixing TiO_2_ with the selected organosilicon modifier was carried out in accordance with the data contained in the publication [36]. In the modification process, TiO_2_ systems were obtained containing 0.5% or 1.5% *w*/*w* modifiers in relation to dry TiO_2_, respectively (see Table 1).

### 2.5. Fabrication of Filaments

#### 2.5.1. Preparation of Granulates

The polymer and the filler were homogenized using a laboratory two-roll mill ZAMAK MERCATOR WG 150/280. A portion of 900 g PLA Ingeo™ 2003 D was mixed with 100 g of modified TiO_2_, until the final concentration of the additive of 10% *w*/*w* was reached. The mixing was performed at the roll temperature of 210 °C for 15 min, getting to full homogeneity of the concentrates. Masterbatch was granulated with a grinding mill SHINI SG-1417-CE and then dried for 24 h at 50 °C.

#### 2.5.2. Extrusion of Filaments

The granules obtained as above were diluted with neat PLA up to the final filler concentrations of 0.5% and 1.5% *w*/*w* upon extrusion molding used for molding of filaments of 1.75 mm in diameter by a single-screw extrusion setup HAAKE Rheomex OS.

### 2.6. 3D Printing (FDM)

Two types of samples for mechanical, impact, microscopic, the measurements of hiding power and WCA tests were printed by FDM using a Creality Ender 3 3D printer: paddles and bars, according to PN-EN- ISO 527−2. Parameters of printing are given in Table 2.

## 3. Results and Discussion

### 3.1. Chemical Characterization of Modifiers

**Figure 1:**
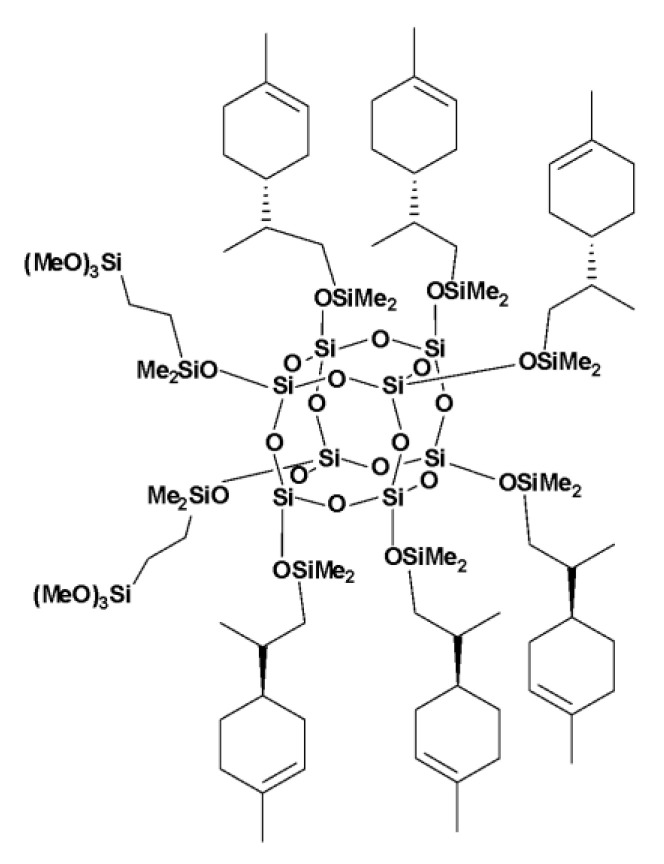
Structure of SS:6LIMO:2VTMOS.

Organosilicon modifiers were obtained by the hydrosilylation reaction of an octahydrosilicate with commercially available olefins. The monofunctional derivative (vinylspherosilicate substituted with eight octadecyl groups) and difunctional derivatives containing octadecyl and trimethoxysilyl or limonene and trimethoxysilyl groups (Figure 1) in various molar ratios were obtained. The progress of the reaction was monitored by FT-IR-ATR analysis until the Si-H bands (at 2141 cm^−1^ and 889 cm^−1^—stretching and bending vibrations, respectively) disappeared. The new derivatives were obtained with a high yield (>97%) and full conversion (>99%), which confirms the efficiency and effectiveness of the hydrosilylation reaction in obtaining functionalized organosilicon compounds. The substrates’ conversion as well as the purity of the products were determined using NMR spectroscopy analysis.

**SS—6Limonen—2VTMOS**

(1,3,5,7,9,11,13,13,15)-hexa(dimethyl((2-(4-methylcyclohex-3-en-1yl)propyl)silyl)-di((trimethoxysilyl)ethyldimethylsiloxyl-pentacyclo[9.5.1.1^3,9^.1^5,15^.1^7,13^]octasiloxane (Figure 1).

^1^H NMR (400 MHz, CDCl_3_): δ (ppm) = 5.36 (s, 6H, ring position 3), 3.55 (s, 18H, OMe), 2.00–1.87 (m, 18H, ring positions 1, 2, 5), 1.78–1.56 (m, 18H, ring positions 2, 5, 8), 1.63 (s, 18 H, -CH_3_ methyl attached to ring position 4), 1.34–1.18 (m, 12 H, ring position 6), 1.11 (d, 3H, produkt alfa-CH_3_), 0.90 (d, 18H, isopropenylmethyl), 0.75–0.69 (m, 6H, isopropenyl-CH_a_H_b_-), 0.59 (s, 4H, SiCH_2_CH_2_Si(OMe)_3_), 0.52–0.44 (m, 6H, isopropenyl-CH_a_H_b_-), 0.13 (s, 36 H, SiMe2);

^29^Si NMR (79.5 MHz, CDCl_3_): δ (ppm) = 12.75 (SiMe_2_), −41.62, (OSi(OMe)_3_), −109.08 (core).

### 3.2. Thermal Analysis (TGA, DSC)

**Figure 2:**
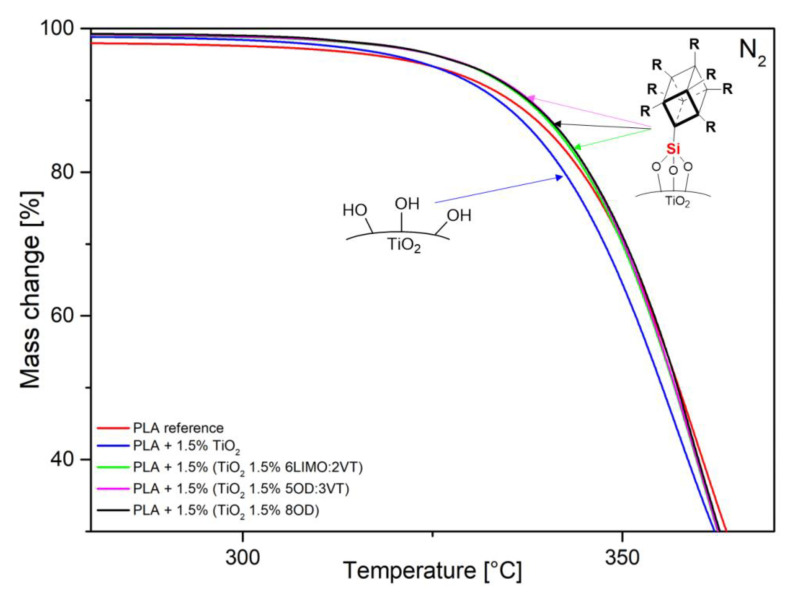
TGA curves under nitrogen atmosphere.

Thermogravimetric analysis (TGA) and differential scanning calorimetry (DSC) were performed for both reference samples—neat polymer (PLA) and PLA with unmodified TiO_2_, as well as for PLA/TiO_2_/organosilicon modifier composites. The tests were carried out for the concentrations of 1.5% wt. TiO_2_ in a polymer matrix and 1.5% wt. of modifier in titanium dioxide. TGA analysis (Table 3, Figure 2) showed that the addition of TiO_2_ slightly lowers the decomposition onset temperature (onset lower by 3.9 °C), which may be due to the presence of Lewis acid centers. Similar effects were described in a work by Xiao Wang et al., who noticed a decrease in the degradation temperature of PLA with the addition of TiO_2_ compared to a pure polymer. TiO_2_ shows a catalytic effect in the thermal degradation of polylactide [36]. The addition of organosilicon modifiers capable of interacting with the TiO_2_ surface causes a change in acidity and deactivation of the catalytic properties of TiO_2_, therefore a slight effect of modified titanium dioxide on polyester decomposition is observed.

**Figure 3:**
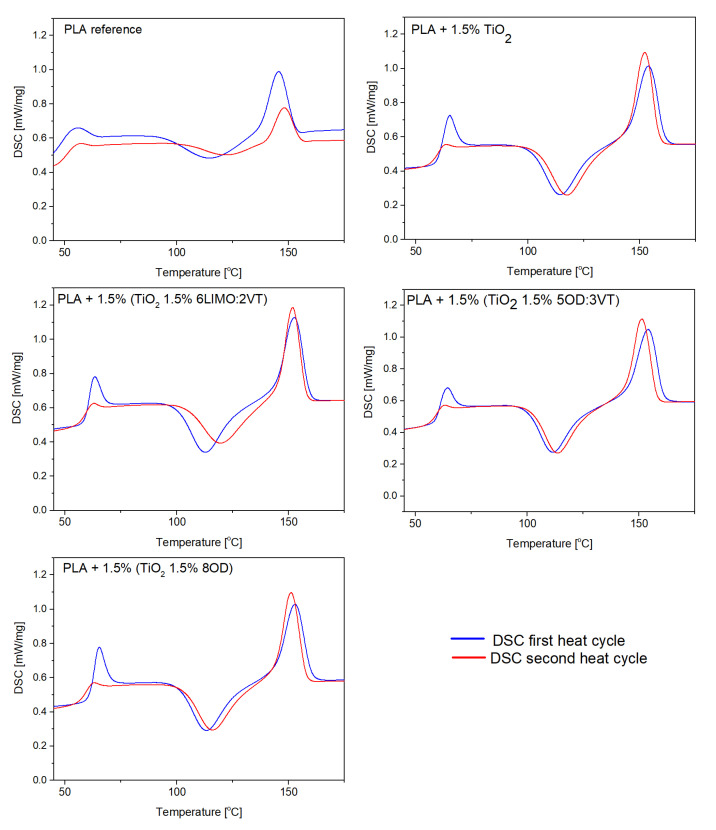
DSC curves of PLA/TiO_2_/modifier composites.

Based on DSC analysis, phase transitions (glass transition temperature T_g_, cold crystallization temperature T_cc_, melting point T_m_) of neat PLA, polylactide with the addition of titanium white TiO_2_ and PLA/TiO_2_/organosilicon compound composites were determined. Figure 3 shows the DSC curves of the tested materials during the first and second heating cycles. Each curve (Figure 3) shows three characteristic temperature ranges, i.e., 60–66 °C (T_g_), 110–130 °C (T_cc_) and 150–155 °C (T_m_).

In the DSC curves (Figure 3) of the composite samples, a large glass transition peak is observed in the first cycle, which is probably the result of a high proportion of the amorphous phase of the polymer. All data are collected in the Table 4. The addition of TiO_2_ or TiO_2_/modifier to the PLA matrix did not significantly affect the glass transition effect. However, DSC analysis showed that the addition of the filler significantly influenced the cold crystallization (T_CC_), which is visible on the curves for both the first and second cycle. For composite systems, a clear, sharp peak in the range of 110–130 °C appears, indicating the occurrence of cold crystallization. The pure PLA curve is characterized by a broad T_cc_ signal, which occurs for semi-crystalline materials with a low crystallization rate. The analysis shows that the fillers TiO_2_ or TiO_2_ with organosilicon additive show the properties of nucleation in the polymer matrix. Both the addition of inorganic filler and TiO_2_ with organosilicon modifiers do not affect the glass transition temperature or melting point compared to pure PLA polymer.

### 3.3. Images of the PLA/TiO_2_ Composites Surface

**Figure 4:**
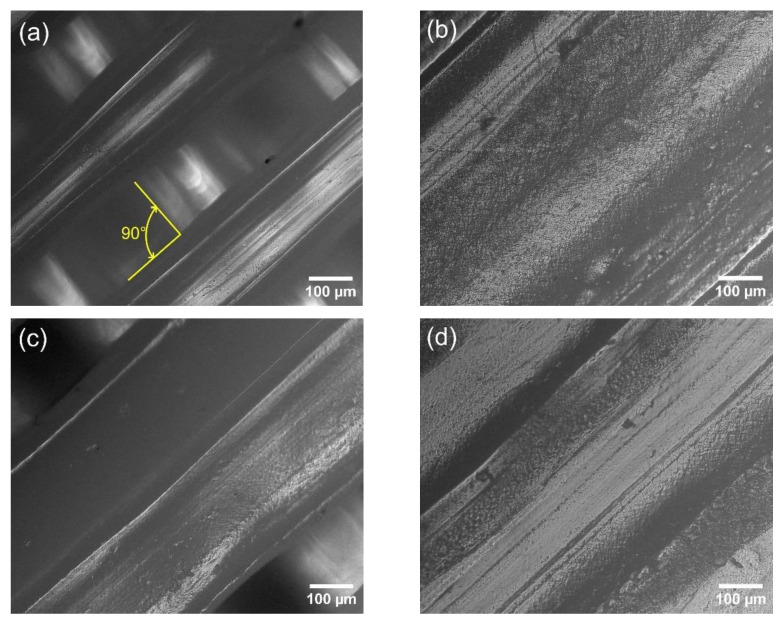

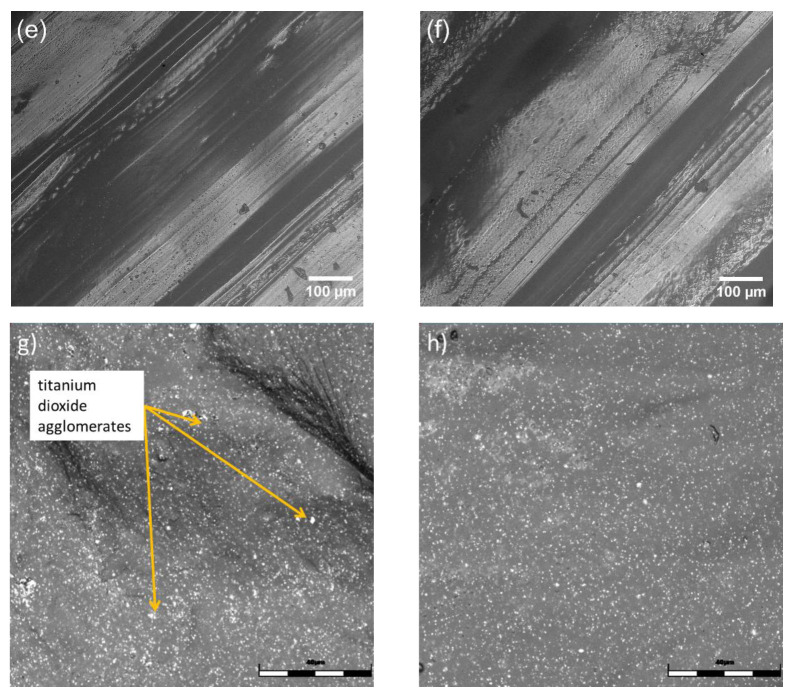
The filaments surface of: (**a**) PLA + 0.5% TiO_2_, (**b**) PLA + 1.5% TiO_2_, (**c**) PLA + 0.5% TiO_2_ + 1.5% 8OD, (**d**) PLA + 1.5% TiO_2_ + 1.5% 8OD, (**e**) PLA + 1.5% TiO_2_ + 1.5% 5OD:3VT, (**f**) PLA + 1.5% TiO_2_ + 1.5% 6Limo:2VT, (**g**) PLA + 1.5% TiO_2_ (image from a confocal microscope), (**h**) PLA + 1.5% TiO_2_ + 1.5% 8OD (image from a confocal microscope).

Figure 4 shows optical microscope images of the cross-section and surface of the filament of the selected samples. The observation of the image shows that the paths of the successive layers are arranged at an angle of 90 degrees (Figure 4a). The surface of the filaments with additions of modifiers differs from the reference samples. The surface of the test samples with the addition of 1.5% modifier 8OD and 6Limo:2VT is less smooth than the 5OD:3VT. The results of mechanical tests indicate that the samples with the 6Limo:2VT modifier has higher mechanical properties and it may be related to a more irregular surface and to a higher surface layer adhesion. In addition, agglomeration of particles was noted in the samples PLA + 1.5% TiO_2_ and PLA + 1.5% TiO_2_ + 1.5% 8OD, which affects the properties of these materials.

### 3.4. Water Contact Angle Analysis (WCA)

#### 3.4.1. WCA of Modified TiO_2_

**Figure 5:**
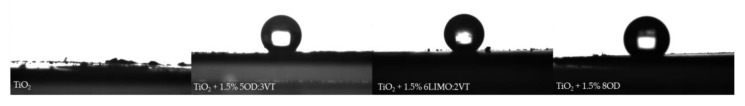
Images of water droplets during sessile drop analysis.

The analysis of the water contact angle (WCA) was carried out for unmodified TiO_2_, as well as TiO_2_ with the addition of organosilicon compounds, to determine the effect of the modifiers on the hydrophilic-hydrophobic properties of the composites surface (Table 5, Figure 5). The test samples in the form of pellets were obtained on a laboratory press under a load of 120 MPa for 5 min.

The surface of titanium dioxide is highly polar, causing the water droplets to be completely absorbed during the measurement. A similar effect is observed for the modification of TiO_2_ with 0.5% organosilicon compound. However, TiO_2_ modified with 1.5% spherosilicate derivatives are characterized by contact angle values above 135°, which indicates the superhydrophobic nature of the filler. The addition of a modifier containing nonpolar alkyl groups, such as octadecyl, changes the surface properties of the filler and reduces the affinity of the tested surface to water. The limonene derivative, from the group of terpenes, in which the only regions with a weak negative partial charge are C=C double bonds, indicates that these charges are too weak to interact with water molecules, resulting in strong hydrophobic properties. Increased affinity to water in the case of 0.5% of the modifier additive is due to the effect of incomplete coverage of the surface hydroxyl groups interacting with the modifier particles. The presence of free -OH groups causes the filler to absorb water. A concentration of only 1.5% gives the desired effect towards the hydrophobicity of the materials.

#### 3.4.2. WCA of PLA/TiO_2_/Organosilicon Compound Composites

**Figure 6:**
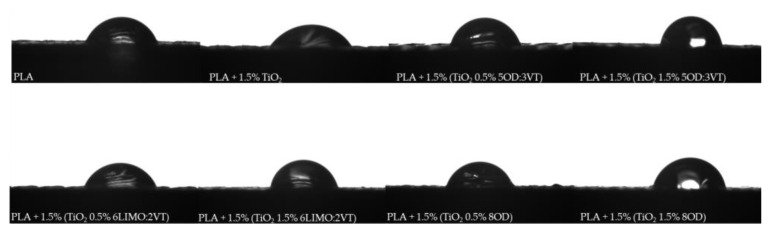
Images of water droplets during sessile drop analysis for composites.

The analyses were carried out for two reference systems, i.e., neat polylactide and a PLA composite with unmodified TiO_2_, as well as for samples with chemically modified titanium dioxide. All data are collected in the Table 6. For the neat PLA surface, a maximum value of 71.3° contact angle was reached, an addition of 1.5 wt.%. unmodified TiO_2_ lowers the contact angle by 4.5° due to the hydrophilic nature of the filler itself. For the PLA + 1.5% TiO_2_ sample, a higher measurement of standard deviation of ±5.5° is also noticeable. This may be due to the formation of titanium dioxide agglomerates in the polymer matrix (see Figure 4g). The highest increase in WCA was observed for samples with 1.5% organosilicon modifier content, of which the greatest improvement in hydrophobic properties was obtained for the modifier (5OD:3VT). Octadecyl groups, due to their non-polarity and chain length, determine the change of the contact angle value, while the methoxy groups may interact with the filler, which increases the compatibility of both components. For white-modified composites with an organosilicon modifier content of 0.5% by weight no change in the value of the contact angle was observed. Figure 6 shows photos of contact angle measurements for modified composites.

### 3.5. Mass Flow Rate (MFR)

**Figure 7:**
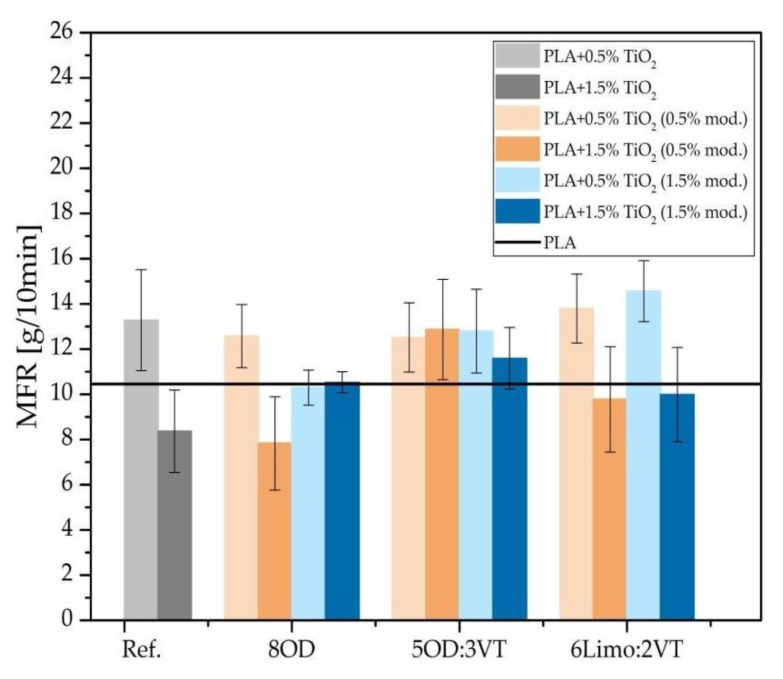
Results of the Mass Melt Flow Ratio Measurements.

Mass flow rate measurements were carried out for the neat PLA, for the reference PLA/TiO_2_ sample and for the PLA/TiO_2_/organosilicon modifier samples. As shown in the Figure 7, the base value for PLA was 10.5 g/10 min at 210 °C. The values for PLA systems with 0.5% filler was 13.5 g/10 min, while increasing the polymer filling to 1.5% TiO_2_ resulted in a reduction of this value in relation to neat polymer to about 8 g/10 min. For the modified systems, a similar effect of MFR increase was observed at lower titanium dioxide fillings. Higher filler content resulted in a slight decrease in these values, but for most systems the decrease was lower than in the case of the reference sample—it is mostly affected by the addition of modifiers. In the case of unmodified titanium dioxide, a decrease in the MFR parameter is visible, which indicates an increase in the viscosity of the polymer, caused by a larger share of filler. In the case of modified systems, deviations from this rule are observed, for the 8OD system, a smaller decrease in viscosity is visible for systems with a higher content of modifier, regardless of the amount of filler used. For the 6LIMO2:2VT system, a slight increase in MFR parameters is observed for systems containing 0.5% of modified TiO_2_ compared to the reference sample with the same filler content. The best results were obtained for the 5OD:3VT system, where there is an increase in the MFR value for all modified systems, including those containing 1.5% of the modifier; it is an increase of over 50% compared to the reference sample with the same filling of the system, which proves the plasticizing effect of this modifier.

### 3.6. Measurement of the Relative Opacity

**Figure 8:**
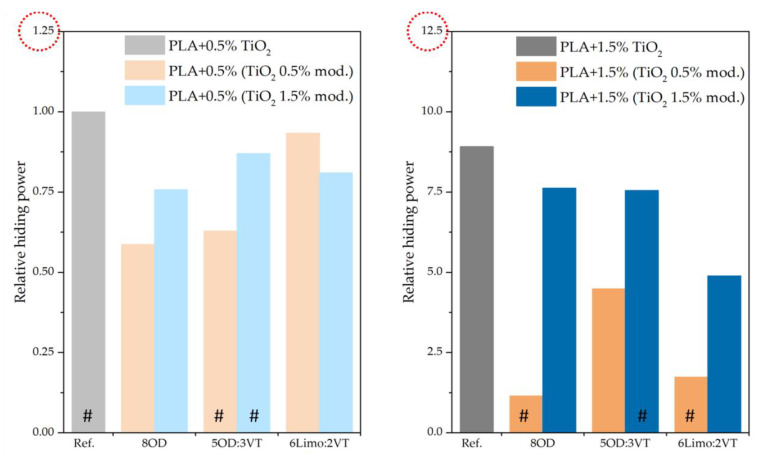
Results of the relative hiding power.

**Figure 9:**
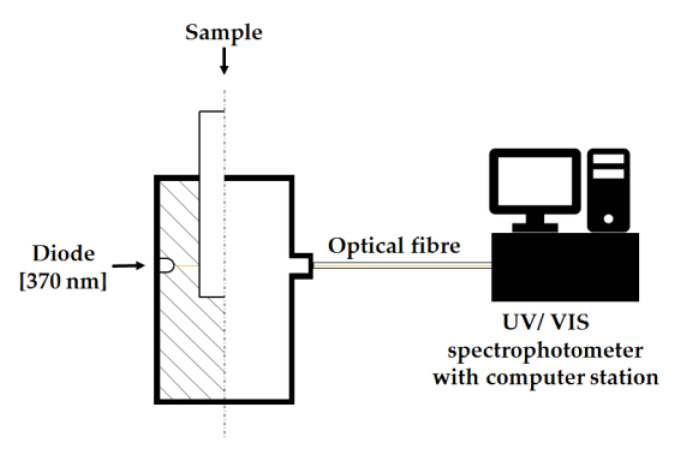

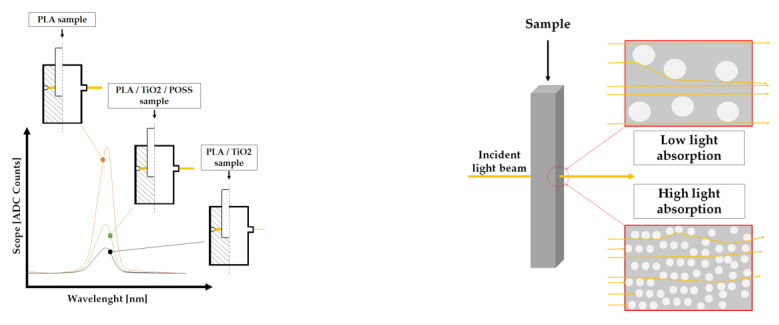
Measurement scheme of Relative hiding power (RHP).

Relative hiding power (RHP) was measured for bars printed on a FDM 3D printer and results are presented in the Figure 8. The PLA/TiO_2_ system was used as a reference. The reference, as well as other samples with modified TiO_2_ of organosilicon compounds, were tested. As expected, RHP increases with an increasing concentration of titanium dioxide in the polymer. In the graphs presented in Figure 9, a decrease in the relative hiding power for systems modified with organosilicon compounds is observed, the highest RHP value for the 8OD sample and the lowest one for the 5OD:3VT sample. The loss of opacity is most likely due to the formation of TiO_2_ agglomerates in the presence of modifiers resulting from the interaction of free hydroxyl groups of the filler, which did not interact with the functional groups of the modifier. This is consistent with microscopic observations (no visible agglomerates on the surface of the composite). For most modifiers, an increasing trend in RHP can be observed with the concentration of the modifier in the filler (a decrease in the amount of surface hydroxyl groups of the filler as a result of attaching more modifier molecules to them). This relationship is observe for all PLA samples with 1.5% of modified titanium dioxide and 0.5% of TiO_2_ content modified with 8OD and 5OD:3VT compounds. However, the sample printed from the composite PLA + 0.5% TiO_2_ 6Limo:2VT shows the opposite tendency. Based on the above diagram, it can be concluded that the addition of modifiers in the tested composites negatively affects the dispersion of titanium white in the PLA polymer matrix.

### 3.7. Tensile Strength and Impact Strength Tests

**Figure 10:**
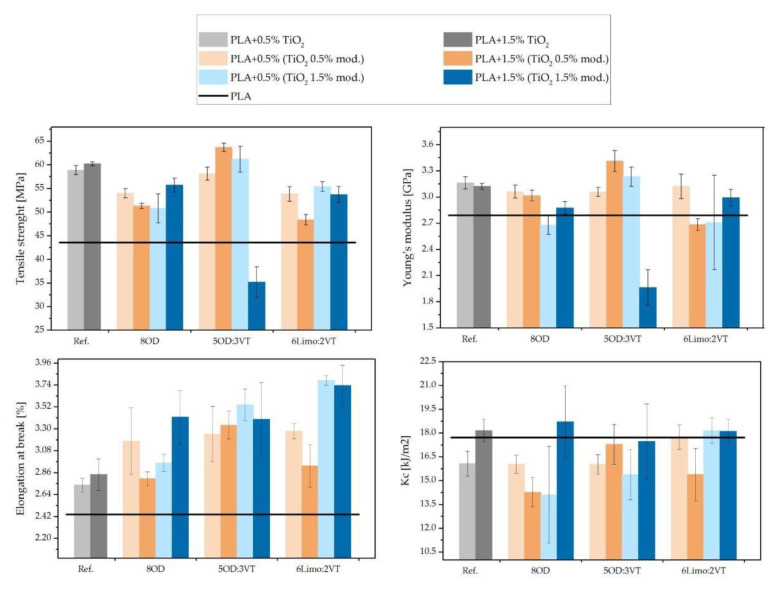
Tensile strength, Young’s modulus, elongation at break and impact strength (Kc) of tested sample.

The results of tensile strength, Young’s modulus, elongation at break and K_c of tested sample variants are presented in the Figure 10. Generally, the addition of 0.5% or 1.5% TiO_2_ significantly enhances the mechanical properties of neat PLA, as the tensile strength increased from 43 MPa to around 60 MPa, while Young’s modulus rose from 2.8 GPa to approx. 3.1 GPa. Moreover, the addition of TiO_2_ not only causes the improvement of tensile strength and Young’s modulus, but also the simultaneous rise of elongation at break value, as it increased slightly from 2.45% to 2.74% and 2.84%, for 0.5% and 1.5% TiO_2_, respectively. Additionally, the influence of TiO_2_ content (0.5% vs. 1.5%) is negligible in tensile and Young’s modulus results, but samples containing 1.5% TiO_2_ had considerably higher elongation and K_c, as compared to samples containing 0.5% TiO_2_. Furthermore, the effect of the addition of modifiers was also investigated. As can be seen in Figure 10, in most cases the addition of selected modifiers moderately worsens strength properties, but simultaneously the addition of 0.5% or 1.5% 8OD caused slight decrease of tensile properties and Kc, but, on the other hand, improves elongation at break value. The addition of 5OD:3VT turned out to be most advantageous in terms of improvement of mechanical properties. However, the sample containing 1.5% TiO_2_ and 1.5% 5OD:3VT exhibited a drastic drop of tensile strength. Samples containing the 6Limo2VT modifier have very similar strength properties to those with the 8OD addition, but are characterized by better elongation and K_c. The change in mechanical properties for the PLA/TiO_2_/5OD:3VT system in relation to the reference PLA/TiO_2_ and PLA system is caused by the plasticizing effect of the modifier on the composite. Meanwhile, the observed deterioration of the tensile strength and impact values for the remaining samples indicates that the modifiers cause agglomeration of the filler, which is also noticeable on microscopic photos.

### 3.8. Flexural Tests

**Figure 11:**
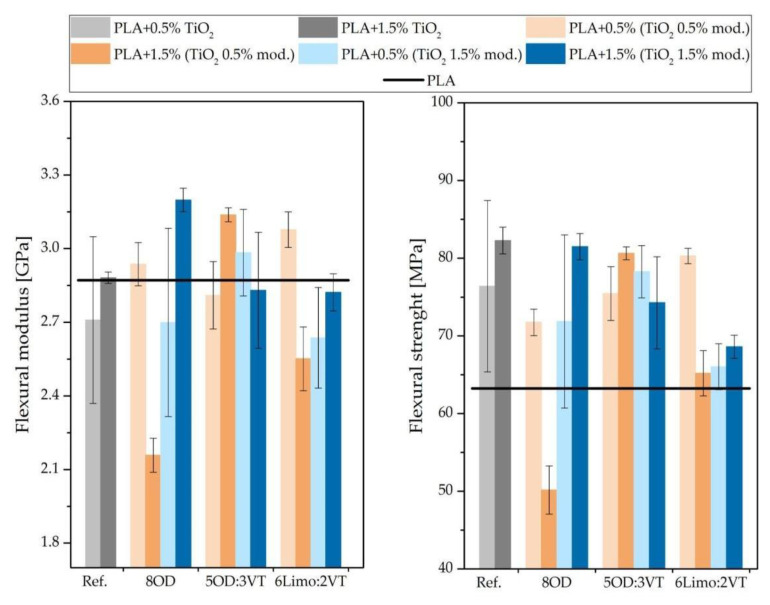
Flexural modulus and flexural strength tested sample.

Flexural tests were conducted following the ISO standard 178:2006 and carried out on composites conditioned at room temperature. Seven bars from each series were used for test (see Figure 11). Maximum deflection measured according to the previously mentioned standard was 6 mm. The samples did not fracture during the flexural strength test. As can be seen in the chart above, in almost all composites containing TiO_2_ the tensile strength properties in relation to unmodified PLA increased, only one of the obtained composites, PLA + 1.5% (TiO_2_ 0.5% 8OD) shows a decrease in value of flexural strength. As the concentration of unmodified TiO_2_ in the polymer increases, the strength of the composite increases. The increase in flexural strength in relation to the reference sample containing 0.5% TiO_2_ was observed for polylactide with the addition of 0.5% modified titanium white 6Limo:2VT (increased by 4.85%) and 1.5% 5OD:3VT (increased by 2.38%). Samples with a higher content of modified TiO_2_ do not show an increase in the value of flexural strength. The modification of TiO_2_ with a small amount of organosilicon derivatives significantly increases the flexural modulus. Such a property was observed for all three additives; however, it depends on the concentration of modified TiO_2_ in the polymer and on the amount of modifier added to TiO_2_. The highest increase in the modulus of elasticity was observed for the PLA sample + 1.5% (TiO_2_ 1.5% 8OD) when compared to the reference sample PLA + 1.5% unmodified TiO_2_ (which, despite the addition of titanium dioxide, does not differ significantly from neat PLA).

The modulus of elasticity determined for the PLA sample + 1.5% (TiO_2_ 0.5% 8OD) is the lowest value among all tested composites and is ~28% lower in relation to polylactide without the additive, which proves the modifier’s influence on the improvement of the elasticity of the composite.

## 4. Conclusions

As a part of the research, TiO_2_ modified with spherosilicate derivatives were obtained, which were then used as pigmentation fillers in PLA-based composites. The TGA experiments showed the effect of surface hydroxyl groups on the reduction of the thermal stability of the filler. The addition of organosilicon substituting for -OH groups eliminates these changes. The effect of the additive on the change of the surface character and obtaining hydrophobic properties for the filler was observed. At higher concentrations of the modifier, the effect of increasing the value of the contact angle is also observed for composites. In MFR measurements, the effect on a significant increase in the flow rate is observed at higher concentrations of the filler and modifier. For the lower values, such an effect was not present. It was observed that the additives significantly improved the mechanical properties in relation to neat PLA, while in relation to the reference TiO_2_ sample, the improvement was mainly observed for the 5OD:3VT system. Microscopic photos confirm that agglomerates formation is lowest for this system.

## Figures and Tables

**Table 1:** Contents of PLA/TiO_2_/modifiers composites.

No	Code	Organosilicon Compound	Amount of Organosilicon Compound [%]	Amount of TiO_2_ [%]
1	PLA	-	-	-
2	PLA + 0.5% TiO_2_	-	-	0.5
3	PLA + 1.5% TiO_2_	-	-	1.5
4	PLA + 0.5% (TiO_2_ 0.5% 8OD)	SS:8OD	0.5	0.5
5	PLA + 0.5% (TiO_2_ 1.5% 8OD)	SS:8OD	1.5	0.5
6	PLA + 1.5% (TiO_2_ 0.5% 8OD)	SS:8OD	0.5	1.5
7	PLA + 1.5% (TiO_2_ 1.5% 8OD)	SS:8OD	1.5	1.5
8	PLA + 0.5% (TiO_2_ 0.5% 5OD:3VT)	SS:5OD:3VTMOS	0.5	0.5
9	PLA + 0.5% (TiO_2_ 1.5% 5OD:3VT)	SS:5OD:3VTMOS	1.5	0.5
10	PLA + 1.5% (TiO_2_ 0.5% 5OD:3VT)	SS:5OD:3VTMOS	0.5	1.5
11	PLA + 1.5% (TiO_2_ 1.5% 5OD:3VT)	SS:5OD:3VTMOS	1.5	1.5
12	PLA + 0.5% (TiO_2_ 0.5% 6Limo:2VT)	SS:6LIMO:2VTMOS	0.5	0.5
13	PLA + 0.5% (TiO_2_ 1.5% 6Limo:2VT)	SS:6LIMO:2VTMOS	1.5	0.5
14	PLA + 1.5% (TiO_2_ 0.5% 6Limo:2VT)	SS:6LIMO:2VTMOS	0.5	1.5
15	PLA + 1.5% (TiO_2_ 1.5% 6Limo:2VT)	SS:6LIMO:2VTMOS	1.5	1.5

**Table 2:** Process parameters for sample printing.

Properties	Values
Layer height	0.2 mm
First layer height	0.2 mm
Number of shells	2
Top and bottom layers number	3
Nozzle diameter	0.4 mm
Infill density	100%
First layer speed	20 mm/s
Printing speed	60 mm/s
Bed temp.	60 °C
Extruder temp.	210 °C

**Table 3:** Results of thermogravimetric analysis under N_2_ atmosphere.

Code	Onset Temperature [°C]
PLA reference	342.6
PLA + 1.5% TiO_2_	338.7
PLA + 1.5% (TiO_2_ 1.5% 6LIMO:2VT)	343.0
PLA + 1.5% (TiO_2_ 1.5% 5OD:3VT)	343.0
PLA + 1.5% (TiO_2_ 1.5% 8OD)	343.7

**Table 4:** Results of differential scanning calorimetry analysis.

Code	Heat Cycle	Glass Transition (T_g_) [°C]	Crystallization (T_c_) [°C]	Melting (T_m_) [°C]
PLA	first	62.0	124.5	154.5
	second	62.9	127.6	153.9
PLA + 1.5% TiO_2_	first	64.3	114.5	154.0
	second	63.1	117.3	152.3
PLA + 1.5% (TiO_2_ 1.5% 5OD:3VT)	first	63.6	111.4	151.1
	second	62.5	113.6	154.7
PLA + 1.5% (TiO_2_ 1.5% 6LIMO:2VT)	first	63.2	112.5	152.5
	second	62.3	119.3	151.8
PLA + 1.5% (TiO_2_ 1.5% 8OD)	first	65.1	113.3	152.8
	second	62.7	115.7	151.1

**Table 5:** Water contact angle of TiO_2_ [°].

Sample Name	Contact Angle [°]
TiO_2_	0
TiO_2_ 0.5% 5OD:3VT	0
TiO_2_ 1.5% 5OD:3VT	141.4 ± 3.4
TiO_2_ 0.5% 6LIMO:2VT	0
TiO_2_ 1.5% 6LIMO:2VT	138.9 ± 2.9
TiO_2_ 0.5% 8OD	0
TiO_2_ 1.5% 8OD	142.8 ± 3.5

**Table 6:** Water contact angle of composites [°].

Code	Contact Angle [°]
PLA	71.3 ± 3.1
PLA + 1.5% TiO_2_	66.8 ± 5.5
PLA + 1.5% (TiO_2_ 0.5% 5OD:3VT)	71.0 ± 1.7
PLA + 1.5% (TiO_2_ 1.5% 5OD:3VT)	82.5 ± 1.1
PLA + 1.5% (TiO_2_ 0.5% 6LIMO:2VT)	71.6 ± 1.0
PLA + 1.5% (TiO_2_ 1.5% 6LIMO:2VT)	76.6 ± 3.8
PLA + 1.5% (TiO_2_ 0.5% 8OD)	70.8 ± 1.6
PLA + 1.5% (TiO_2_ 1.5% 8OD)	77.4 ± 2.1
